# Radiotherapy induces an immediate inflammatory reaction in malignant glioma: a clinical microdialysis study

**DOI:** 10.1007/s11060-016-2271-1

**Published:** 2016-09-23

**Authors:** Pedram Tabatabaei, Eward Visse, Per Bergström, Thomas Brännström, Peter Siesjö, A. Tommy Bergenheim

**Affiliations:** 1 Department of Clinical Neuroscience, Neurosurgery, Umea University, 901 85 Umeå, Sweden; 2 Department of Medical Bioscience, Umeå University, 901 85 Umeå, Sweden; 3 Department of Radiation Science, Umeå University, 901 85 Umeå, Sweden; 4 Department of Clinical Science, Lund University Hospital, 221 85 Lund, Sweden

**Keywords:** Cytokine, Glioblastoma, Radiotherapy, Microdialysis, Inflammation

## Abstract

The knowledge of response to radiation in the immuno-microenvironment of high grade gliomas is sparse. In vitro results have indicated an inflammatory response of myeloid cells after irradiation. Therefore, microdialysis was used to verify whether this is operative in tumor tissue and brain adjacent to tumor (BAT) after clinical radiotherapy of patients with high grade glioma. Stereotactic biopsies and implantation of microdialysis catheters in tumor tissue and BAT were performed in eleven patients with high-grade glioma. The patients were given daily radiation fractions of 2–3.4 Gy. Microdialysis samples were collected before radiotherapy and during the first five days of radiation. Cytokines, glucose metabolites, glutamate and glycerol were analyzed. Immunohistochemistry was performed to detect macrophages (CD68) and monocytes (CD163) as well as IL-6, IL-8 and MCP-1. A significant increase of IL-8, MCP-1 and MIP-1a were detected in tumor tissue already after the first dose of radiation and increased further during 5 days of radiation. IL-6 did also increase but after five fractions of radiation. In BAT, the cytokine response was modest with significant increase of IL-8 after third dose of radiation. We found a positive correlation between baseline IL-8 and IL-6 microdialysis levels in tumor tissue and survival. Glucose metabolites or glycerol and glutamate did not change during radiation. In all tumors staining for macrophages was demonstrated. IL-6 was found in viable tumor cells while MCP-1 was demonstrated in macrophages or tumor matrix. Our findings suggest that radiation induces a rapid enhancement of the prevailing inflammation in high-grade glioma tissue. The microdialysis technique is feasible for this type of study and could be used to monitor metabolic changes after different interventions.

**Electronic supplementary material:**

The online version of this article (doi:10.1007/s11060-016-2271-1) contains supplementary material, which is available to authorized users.

## Introduction

Glioblastoma multiforme is the most common primary brain tumor and despite recent advances in the treatment the median time of survival is no >16 months after diagnosis [1]. During the last decade the importance of the tumor microenvironment of gliomas has gradually been acknowledged [2–4]. The tumor microenvironment in the brain contains an array of cells as microglia, reactive astrocytes, reactive neurons, endothelial cells, blood derived myeloid and lymphoid cells, and stromal cells as mesenchymal stromal cells (MSC) and cancer associated fibroblast (CAF). Both immune and stromal cells induce an ongoing inflammation within CNS tumors and the controversy has centered upon whether this is in favor of the tumor or of the patient [5]. Accumulated data show that myeloid cells are the most common inflammatory cells in infiltrating human gliomas and thus of crucial importance for the microenvironment of the tumor [6]. Myeloid cells, as dendritic cells, microglia and macrophages, can both boost or inhibit innate and adaptive immune responses against tumors by releasing pro or anti-inflammatory cytokines [4, 7, 8]. Apart from direct modulation of anti-tumor immunity, myeloid cells and other cells in the tumor microenvironment can promote tumor growth by tuning of angiogenesis, proliferation, metabolism and resistance against chemo- and radiotherapy [2, 9, 10].

In this scenario one important issue is how the immune-microenvironment of the CNS affects and is affected by different treatment modalities. Radiation therapy is one of the pillars of malignant glioma treatment since several decades. The challenge of radiotherapy is to ablate the tumor without excessive damage to the surrounding tissue. It is well known that following radiation exposure there is both parenchymal and vascular damage leading to a radiation induced cell death, involving not only tumor cells, but also oligodendrocytes, neural progenitors and endothelial cells [11, 12]. Recent research has also demonstrated that the effect of radiation is not only dependent on direct killing of tumor cells or tumor vessels but also by inflammatory and immune secondary effects [13]. While microglia and macrophages are more resistant to irradiation than other cells, they react by increasing production of reactive oxygen species (ROS) and nitric oxide synthase (NOS). This together with release of damage associated molecular patterns (DAMP) from damaged cells, induce inflammatory transcription factors as NFkB and RelB leading to release of inflammatory cytokines and chemokines. These can induce angiogenesis, edema and tissue damage, but will also recruit more inflammatory cells by chemotaxis and thus alter the immune-microenvironment [11, 14–16]. The changes in cellular composition at the site of radiation mainly result in an increased number of reactive astrocytes, macrophages and microglia [13, 17]. Accumulated evidence from experimental in vitro and vivo studies has provided indirect evidence that radiation of glioma cells or glioma tissue leads to an increase in the secretion of inflammatory cytokines [11, 18].

A better understanding of the inflammatory response in glioma after radiotherapy would be helpful in the development and improvement of novel treatment strategies, such as glioma immunotherapy. But, also in order to maximize tumor cell killing and avoid collateral damage to normal cells. Although circumstantial evidence has indicated that radiotherapy induces the release of inflammatory mediators, no direct in situ proof of this has been presented. For this purpose we have investigated the effects of fractionated radiotherapy on several cytokines involved in the immune and inflammatory system of glioblastoma by the use of microdialysis. As recent research has pointed to a close correlation between metabolism and inflammation [19, 20], we have additionally studied whether inflammation might have any relationship to the metabolic state of the tumor, to cell damage markers, or to clinical outcome.

## Methods

### Patients

Eleven patients were included in the study. All patients had a radiological suspicion of high-grade astrocytoma. The tumors were found not suitable for debulking surgery, and thus a stereotactic serial biopsy was performed for morphological diagnosis. Ten of those patients had a histologically verified glioblastoma, whereas one of the patients had anaplastic astrocytoma (WHO grade III). There were eight men and three females with a mean age of 63 years (range 50–81). The mean survival of the patients after diagnosis was 6.3 months. This series of patients has previously been reported investigating the metabolomic response to radiotherapy [21]. The local ethics committee in Umeå approved the study, and all patients gave their informed consent to participate in the study.

### Surgery

Stereotactic serial biopsies were performed under general anesthesia and with the aid of a Leksell stereotactic frame (Elekta, Stockholm, Sweden). After mounting of the frame a stereotactic CT was performed for target calculations. The method used for stereotactic introduction of the microdialysis catheters have been described in detail earlier [22]. Frozen sections confirmed the diagnosis before the microdialysis catheters were stereotactically introduced. There were two catheters placed, one catheter in the biopsy target area, within the radiologically defined tumor, and a second catheter in brain adjacent to tumor (BAT), approximately 10 mm outside the contrast-enhancing tumor. As a reference, one catheter was put in the abdominal subcutaneous tissue (SC). The accurate location of the intracranial catheters was confirmed on postoperative CT performed for dose planning. The microdialysis equipment was fixed in the head dressing allowing the patient to move freely at the neurosurgical ward.

### Postoperative care and radiotherapy

All the patients recovered and were monitored for the first 24 h after surgery at the neurosurgical intensive care unit. The patients received betamethasone as perioperative routine and blood glucose was kept under 7 mmol/L. Eight patients were given conventional radiotherapy with daily 2 Gy fractions up to 60 Gy. The volume included the contrast enhanced tumor mass with a 2.5 cm margin. The irradiation was started within 2–5 days after biopsy. Three elderly patients in a poor general condition were given a hypofractionated treatment with 3 Gy × 13 (two cases) or 3.4 Gy × 10 (one case). The irradiation was given randomly at daytime between 8 am and 4.15 pm in all patients.

### Microdialysis and analysis

The intracranial microdialysis catheters had 100-mm long shafts with an outer diameter of 0.9 mm and a semipermeable membrane that was 10 mm long, while the subcutaneous reference catheters had 60 mm shafts with a 30 mm long membrane. The semipermeable membrane was made of polyamide and had a 100 kDa cut-off and an outer diameter of 0.6 mm (CMA71; CMA Microdialysis, Stockholm, Sweden). To enable visualization of the catheters on CT, the catheters had a small gold tip. The catheters were connected to a microinfusion pump with a 2.5-mL syringe, set at a flow rate of 0.3 µL/min (CMA 106 or CMA 107; CMA Microdialysis). All catheters were perfused with a Ringer solution (Perfusion fluid T1; CMA Microdialysis) mixed with Dextran (30 g Dextran 60 1000/mL) to prevent microfiltration [23]. The microdialysis ran for at least 20 h before the first dose of radiation and continued during first 5 days of radiation and then additionally for at least 20 h. The microdialysis samples were collected in microvials every second hour, thereafter frozen and kept at −80 °C until analyzed.

### Analysis of glucose metabolites, glycerol and glutamate

For analysis of glucose metabolites, glycerol and glutamate, we selected samples, usually two, during a time span of 4 h between midnight to six in the morning. These samples were thus fasting samples. All samples were analyzed using the CMA 600 analyzer (CMA Microdialysis). The CMA 600 analyzer uses enzymatic reagents and colorimetric measurements of the microdialysis samples [24]. A mean value of these samples were thereafter calculated and used in the statistical analysis.

### Analysis of cytokines

Cytokines were analyzed from samples collected the night before radiotherapy started, as baseline, and thereafter from samples collected during the same hours, after the first, third and fifth fractions of radiation. To analyze the cytokine content of the microdialysis samples, a Cytokine Bead Array (CBA, BD, Stockholm) was performed according to the manufacturers’ recommendations. Flex-sets for the following human cytokines were used: IL-4, IL-6, IL-8, IL-10, TNF-α, IFN-γ, GM-CSF, MCP-1, MIP-1α, MIP-1β. Measurement of IFN-γ and IL-10 were not possible due to technical reasons. Samples were used in 1× or 2× dilution depending on available volume.

### Immunohistochemistry

Tissues for histopathological studies were immersion-fixed in 4 % paraformaldehyde in 0.1 M Na phosphate, pH 7.4 and then paraffin-embedded. From blocks of the stereotactic biopsies 4 µm thick sections were cut using a sliding microtome and mounted on Superfrost™ slides (Thermo Fisher Scientific, Hägersten, Sweden). Slides were stained with Haematoxylin/Eosin and immunostained according to the manufacturer’s recommendations using the Benchmark Ultra (Ventana medical systems Inc, Illkirch, France). The sections for immunostaining were preincubated for 30 min in 3 % H_2_O_2_ in methanol and then heated in 0.5 M citrate buffer (pH 6.0) for 5 min in a microwave oven. The following primary antibodies were used: anti-GFAP (code Z 0334; Dakocytomation, Glostrup, Denmark; 1:5000); anti-vimentin (catalog number 790–2917; Ventana medical systems; 1:1); anti-IDH1(R132H) (clone H09; Dianova, Hamburg, Germany; 1:50); anti-Ki-67 (clone 30-9; Ventana medical systems; 1:50); anti-p53 (clone DO-7; Novocastra™, Newcastle-upon-Tyne, England; 1:25); anti-EGFR (clone 3C6; Ventana medical systems; 1:100); anti-phosphohistone-H3 (catalog number 369A; Cell marque, Rocklin, CA, USA; 1:300); anti-human CD31 (clone JC70A; Dako, Glostrup, Denmark; 1:10); anti-human CD68 (clone KP1; Dako; 1:2000; CC1 pretreatment); anti-CD163 (clone 10D6; Novocastra; CC2 pretreatment); anti-MCP1 (catalog number ab9669; Abcam, Cambridge, England; 1:100; CC2 pretreatment); anti-IL6 (catalog number ab6672; Abcam; 1:200; CC1 pretreatment); anti-IL8 (catalog number 17038-1-AP; Proteintech, Chicago, USA; 1:25; CC1 pretreatment). Micrographs were taken with a Olympus BX53 microscope equipped with a DP73 camera (Olympus, Hamburg, Germany) and using the CellSens dimension software (Olympus).

### Statistical analyses

Statistical analyses were performed with Wilcoxon signed-rank test and ANOVA using SPSS. The correlation coefficients and the coefficient of determination (r-square) were calculated and analysed.

## Results

### Patients

There were no complications such as infection, haemorrhage, or increased cerebral edema in association with the microdialysis or the treatment. The patients were mobilized the day after surgery and could move freely within the neurosurgical ward with the apparatus attached to a head cloth.

### Cytokines

**Fig. 1:**
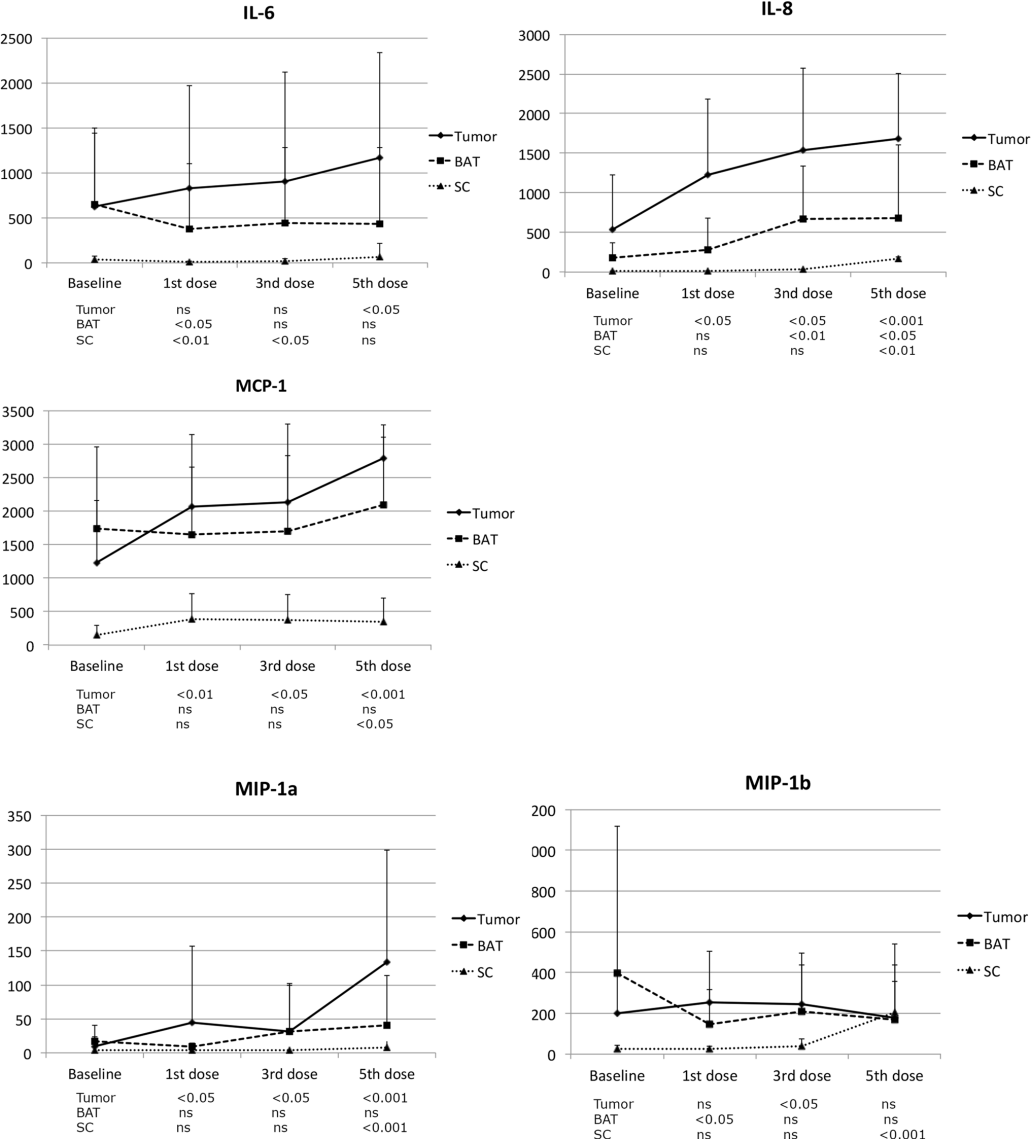
Cytokines assessed by microdialysis in tumor tissue, brain adjacent to tumor (BAT) and abdominal subcutaneous tissue (SC) in 11 patients with malignant glioma before irradiation and after the 1st, 3rd, and 5th dose. The mean (SD) levels of IL-6, IL-8, MCP-1, MIP-1a and MIP-1b are expressed in pg/mL. Statistical analyses comparing the levels after irradiation with base-line with Wilcoxon signed-rank test

The cytokines that were reliably and constantly detected in the microdialysis samples and thus available for statistical analyses are presented in Fig. 1. An increase of IL-6 in the tumor could be observed after the fifth fraction of radiation. In BAT and SC on the other hand, a decrease could be observed already after the first radiation fraction. IL-8 levels in the tumor were significantly increased already within 24 h following the first given fractions of radiation, and increased further thereafter during the whole time of the radiation therapy. The IL-8 concentrations in BAT and SC increased with a delay compared to tumor tissue. MCP-1 concentrations in tumor tissue did also significantly increase already after the first fraction and kept on increasing during the time of radiotherapy. However, a significant increase could only be observed after the fifth fraction of radiation in SC tissue. The increase of MIP 1-a was much similar to MCP-1, with significant increase in tumor during the whole course of therapy, and only after the fifth fraction in SC tissue. Finally, we could also observe a temporary significant increase in MIP 1-b levels in tumor after the third fraction of radiation. The concentration in SC was also increased but not until after the fifth fraction. We found no significant difference between the patients receiving standard dose, 2 Gy, and those receiving 3 and 3.4 Gy.

### Glucose metabolites and markers

Baseline levels for glucose metabolites, glycerol and glutamate were established. The figures are presented in Table 1. There were significant lower glucose and pyruvate levels within the tumor tissue compared to BAT. The levels of glutamate and lactate were on the other hand significantly higher in tumor tissue. Also the lactate-pyruvate ratio was significantly higher within the tumor tissue compared to BAT.

**Table 1:** Glucose metabolites, glutamate and glycerol assessed by microdialysis in tumor tissue, brain adjacent to tumor (BAT) and abdominal subcutaneous tissue (SC) in 11 patients with malignant glioma before irradiation and after the 1st, 3rd, and 5th dose

	Tumor				BAT				SC			
	Day 0	Day 1	Day 3	Day 5	Day 0	Day 1	Day 3	Day 5	Day 0	Day 1	Day 3	Day 5
Glucose (mmol)												
Mean	0.28	0.58	0.22	0.12	1.01	0.81	0.67	0.61	1.97	1.63	1.85	1.88
SD	0.40	0.98	0.30	0.18	1.34	1.14	1.19	0.79	1.29	1.20	0.93	1.28
Lactate (mmol)												
Mean	5.24	5.63	5.44	4.89	3.92	3.74	3.26	3.54	0.75	0.87	0.99	1.03
SD	3.95	3.40	3.37	3.61	2.04	2.17	2.48	2.58	0.76	0.72	0.97	0.86
Pyruvate (µmol)												
Mean	95.08	114.92	112.40	107.20	159.52	134.01	151.15	148.88	61.71	74.07	70.45	78.80
SD	79.93	65.97	74.41	68.71	67.32	69.64	55.73	74.63	42.77	61.15	49.69	68.27
Lactate/pyruvate												
Mean	55.37	48.67	48.40	45.42	22.91	29.56	23.60	16.02	11.78	11.21	13.40	17.12
SD	41.94	34.69	38.89	36.16	20.85	30.98	41.42	115.65	17.16	17.03	15.22	13.95
Glutamate (mmol)												
Mean	42.47	50.48	63.38	66.52	29.97	33.57	44.53	59.48	6.73	8.63	6.59	2.67
SD	50.73	57.16	75.27	79.99	59.06	62.86	82.69	112.22	15.47	17.72	11.61	1.99
Glycerol (mmol)												
Mean	35.46	33.96	29.79	41.69	31.00	22.54	20.56	20.89	112.82	139.36	144.19	130.99
SD	27.23	31.86	18.16	32.85	27.08	20.52	20.38	21.64	82.93	103.86	85.34	142.20

The levels of glucose metabolites were not significantly changed during the course of radiotherapy. Neither were there any changes in the levels of the potential markers for tissue damage, glutamate and glycerol.

### Immunohistochemistry

**Fig. 2:**
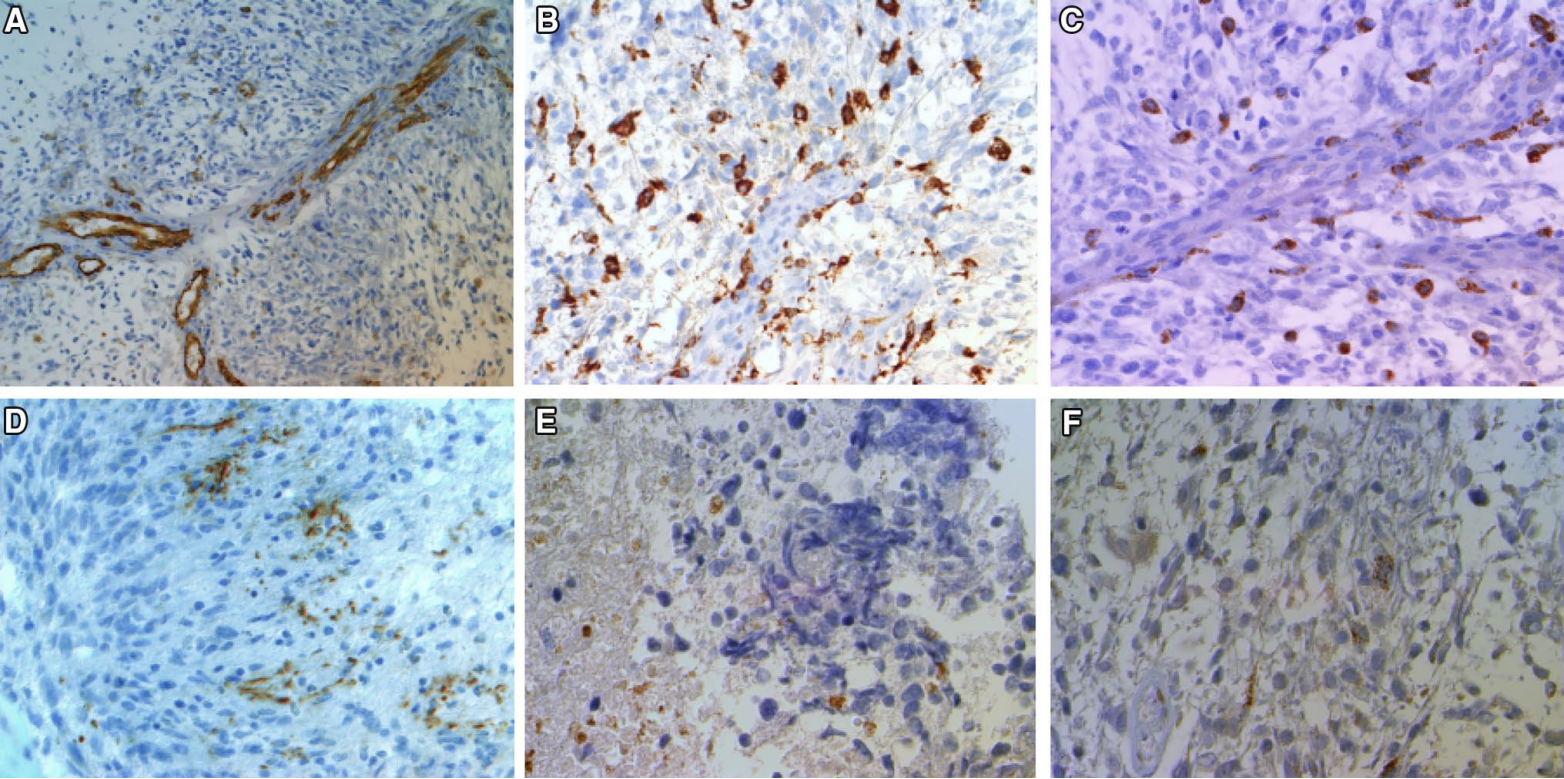
Micrographs from glioblastoma tissue representative for the patients where cytokines were assessed by microdialysis. The sections were stained with CD31 (**A**), CD68 (**B**), CD 165 (**C**), IL-6 (**D**), IL-8 (**E**) and MCP-1 (**F**)

All tumors expressed GFAP (glial fibrillary acidic protein) and vimentin, markers for astrocytic tumors. All patients except two had many cells expressing PHH3 (phosphohistone-H3, a mitosis marker) and high Ki-67 labeling index, indicating a high proliferation with a high mitotic rate in the specimens. This confirms the diagnosis of high grade tumors. The specimens showed an abundance of blood vessels confirmed by the staining of the endothelial marker CD31 (Fig. 2A). Immunoreactivity for IDH1(R132H) was seen in four tumors, three of these being among the eight out of the eleven tumors that had a strong expression of EGFR.

In summary the immunohistochemistry together with the histopathological analysis conclude that all the patients had high grade (grade 3 and 4) astrocytic tumors.

To investigate the presence of infiltrating immunopotent cells such as macrophages and monocytes the specimens were stained for CD68 and CD163. All tumors showed cells that expressed CD163 strongly and all but one showed cells expressing CD68. The macrophages were more often than not seen in close approximation to blood vessels (Fig. 2B, C).

IL-6 was observed at high levels in all specimens. IL-6 could mainly be seen in viable tissue surrounding the necrotic areas (Fig. 2D). Staining could be seen both intra- and extra-cellular. When present, IL-8 and MCP-1 were, on the other hand, observed both in, and in areas surrounding, necrotic parts (Fig. 2E, F). IL-8 could not be detected by immunohistochemistry in five patients while in the other patients it was seen in <25 % of the cells. On the other hand MCP-1 could be found in all patients. These cytokines were observed either in macrophages or in the extracellular matrix.

### Correlations

We found a significant statistical correlation between baseline IL-8 and IL-6 microdialysis levels and survival (r^2^ = 0.28 and r^2^ = 0.13, respectively). However, there was no correlation between the increase following treatment in IL-6, IL-8 and MCP-1 and survival.

## Discussion

There is strong evidence that immune and inflammatory reactivity plays an important role in the regulation of both tumor progression and regression [2, 25]. However, less is known about how different treatment modalities affect the immune and inflammatory-microenvironment in the CNS. Here we demonstrate an increase of IL-8, MCP-1 and MIP-1a intratumorally already within 24 h after the first radiation dose. The increased levels of cytokines even progressed to higher levels during the first days of radiotherapy. Further, we could also observe a delayed increase in the levels of IL-6 and MIP-1b. For IL-6 we could however observe an early decrease in BAT and SC tissue, while IL-8, MCP-1, MIP1-a and MIP1-b showed an increase in BAT and SC tissue, although the increase was somewhat delayed compared to the increase in tumor tissue. MIP 1-b levels were also increased within 24 h after the first radiation dose in BAT. Taken in consideration that insertion of a microdialysis catheter intracerebral induces a decreasing release of inflammatory cytokines during 1–4 days [26, 27] our results strongly indicate that radiation per se induces an intra tumoral inflammatory reaction and an increased production and secretion of cytokines.

The timing of radiation may be one factor that influence the levels of cytokines. However, we do not believe that the varying time points for irradiation was of significant importance since the results rather point towards an accumulation of the inflammatory cytokines over time during the 5 days.

The old concept that the effect of radiation on mammalian cells depends solely on DNA damage has been revised by results demonstrating early release of ROS, NOS, growth factors and pro-inflammatory cytokines [12, 28]. The early NO and ROS production after radiation has been linked to rapid inflammatory changes in target cells but also to structural and inflammatory changes in bystander cells [29–31]. As myeloid cells readily express iNOS they are the most potent producers of NO in the tumor microenvironment after radiation. Interestingly, these reactions have been linked to both collateral damage of non-neoplastic tissue and to initiation of anti-tumor responses [11, 32]. Immunohistochemical studies show that the early response after radiotherapy in high grade glioma is characterized by astrocytic gliosis, vascular proliferation, accumulation of activated microglia, and infiltration of macrophages [14–16]. The gradient of cytokines in our analysis definitely point at an intratumoral source of cytokine release although the BAT area also contributes. Secretomic data from cultured tumor and astrocytic cells clearly indicates that these are capable of producing various cytokines including those highlighted in the present study [33, 34]. Nevertheless accumulated data and our immunohistochemistry results strongly imply that the overwhelming source of secreted inflammatory cytokines emanate from myeloid cell, either infiltrating macrophages or resident microglia and that myeloid cells are the drivers of other early features of reaction as astrocyte gliosis [5, 35, 36].

IL-6 is a pleiotropic cytokine [37, 38] which is amplified and over-expressed in GBM cells both in vivo and in vitro [39]. There is also an increased peripheral and local IL-6 secretion in GBM patients compared with healthy controls [40, 41]. In GBM it has been linked to several features as invasiveness, angiogenesis, radioresistance and short survival [42–44].

IL-8 was first identified as a chemoattractant for granulocytic cells but also has pleiotrophic effects in tumors as direct or indirect induction of angiogenesis, invasiveness, recruitment of bone marrow cells, and maintenance of CSC in the perivascular niche [45–47]. IL-8 could be detected, by immunochemistry, in 50 % of the patients and was found mainly surrounding the necrotic areas of the tumor and around macrophages. However in microdialysis samples it was detected in all patients. The reason for this discrepancy could be that extra and intra cellular proteins as for other cytokines disguise the molecular epitope. The increase in IL-8 and MCP-1 was more rapid than that for IL-6 which could be explained by release of the latter from reacting tumor cells or infiltrating inflammatory cells recruited by the formers.

The immunohistochemical analysis showed a dense infiltration of myeloid cells. All tumors expressed CD163 strongly and all but one expressed CD68. These results are in line with earlier reports where flow cytometric studies have showed that as much as one-third of the cells within gliomas could be microglia and macrophages [6, 48, 49]. Both IL-8 and MCP-1, and to some extent also MIP1-a and MIP1-b, are potent chemoattractants for myeloid and granulocytic cells—and to some extent, lymphoid cells—that will increase influx of inflammatory cells to the radiated tissue. The activated macrophages will secrete or induce the release of, proteolytic enzymes and additional inflammatory cytokines [10, 50–52]. Although the significant longer survival in our patients with high expression of IL-8 indicates that IL-8 could play a beneficial role in the CNS anti-tumoral response the present material is to small to draw any conclusions.

In order to protect themselves against immune surveillance neoplastic cells use several mechanisms to downregulate anti-tumor immunity. With some exceptions inflammation has been shown to repress adaptive and some innate anti-tumor responses by the direct or indirect release of soluble factors as PGE2, TGF-b, NO or by inhibitory ligands as PD-L1, CTLA4 and TIGIT. By indirect means tumor cells can induce secretion of these by induction of monocytic and granulocytic myeloid cells. Despite some reports of enhanced adaptive immune responses, both IL-6 and IL-8 have overwhelmingly been associated with immune suppression through induction of regulatory T cells, myeloid derived suppressor cells [53] or direct effects [54–57]. Although none of the primary emblematic cytokines associated with M (macrophage) or Th (T helper cells) subtypes were increased or detected in our study the pattern recorded more resembles M2 and Th2 subtypes. However, the delicate balance between immune potentiation and immune suppression might depend on the features or extent of inflammation as the therapeutic effects of low dose or non-fractioned irradiation has been shown to depend on immune reactivity [32, 53, 58]. To this end our results show that inflammatory cytokines are increasingly secreted after each fraction of radiotherapy.

## Conclusion

Our findings suggest that irradiation induces a rapid enhancement of the prevailing inflammation in the tumor tissue of human GBMs. The microdialysis technique is feasible for this type of study and could be used to, in vivo, monitor changes in cytokine secretion in high-grade glioma tissue after different interventions.

## Electronic supplementary material

Below is the link to the electronic supplementary material.

Supplementary material 1 (PDF 96 KB)
