# Supplementary material for: Radiotherapy induces an immediate inflammatory reaction in malignant glioma: a clinical microdialysis study
Source: J Neurooncol. 2016 Sep 23;131(1):83–92. doi: 10.1007/s11060-016-2271-1 (PMC5258803; doi:10.1007/s11060-016-2271-1)
Supplement: Supplementary file 1 — Supplementary material 1 (PDF 96 KB) [file 11060_2016_2271_MOESM1_ESM.pdf]

Online Resource 1 for article:

Radiotherapy induces an immediate inflammatory reaction in malignant glioma – a clinical microdialysis study

Pedram Tabatabaei, Eward Visse, Per Bergström, Thomas Brännström, Peter Siesjö, A Tommy Bergenheim

Journal name: Journal of Neuro-Oncology

Corresponding author:

Pedram Tabatabaei

Department of Clinical Neuroscience, Neurosurgery, Umea University, 901 85 Umea, Sweden

Tel:+46-707603239   Mail: pedram.tabatabaie@vll.se

Immunohistochemistry

Tissues for histopathological studies were immersion-fixed in 4% paraformaldehyde in 0.1 M Na phosphate, pH 7.4 and then paraffin-embedded. From blocks of the stereotactic biopsies 4 µm thick sections were cut and mounted on Superfrost™ slides (Thermo Fisher Scientific, Hägersten, Sweden). Slides were stained with Haematoxylin/Eosin and immunostained according to the manufacturer's recommendations using the Benchmark Ultra (Ventana medical systems Inc, Illkirch, France). The sections for immunostaining were preincubated for 30 min in 3% H_2O_2 in methanol and then heated in 0.5 M citrate buffer (pH 6.0) for 5 min in a microwave oven. The following primary antibodies were used: anti-GFAP (code Z 0334; Dakocytomation, Glostrup, Denmark; 1:5000); anti-vimentin (catalog number 790-2917; Ventana medical systems; 1:1); anti-IDH1(R132H) (clone H09; Dianova, Hamburg, Germany; 1:50); anti-Ki-67 (clone 30-9; Ventana medical systems; 1:50); anti-p53 (clone DO-7; Novocastra™, Newcastle-upon-Tyne, England; 1:25); anti-EGFR (clone 3C6; Ventana medical systems; 1:100); anti-phosphohistone-H3 (catalog number 369A; Cell marque, Rocklin, CA, USA; 1:300); anti-human CD31 (clone JC70A; Dako, Glostrup, Denmark; 1:10); anti-human CD68 (clone KP1; Dako; 1:2000; CC1 pretreatment); anti-CD163 (clone 10D6; Novocastra; CC2 pretreatment); anti-MCP1 (catalog number ab9669; Abcam, Cambridge, England; 1:100; CC2 pretreatment); anti-IL6 (catalog number ab6672; Abcam; 1:200; CC1 pretreatment); anti-IL8 (catalog number 17038-1-AP; Proteintech, Chicago, USA; 1:25; CC1 pretreatment). Micrographs were taken with a Olympus BX53 microscope equipped with a DP73 camera (Olympus, Hamburg, Germany) and using the CellSens dimension software (Olympus).
